# Supplementary material for: A safe and non-flammable sodium metal battery based on an ionic liquid electrolyte
Source: Nat Commun. 2019 Jul 24;10:3302. doi: 10.1038/s41467-019-11102-2 (PMC6656735; doi:10.1038/s41467-019-11102-2)
Supplement: Supplementary file 1 — Supplementary Information [file 41467_2019_11102_MOESM1_ESM.pdf]

Supplementary information

A safe and non-flammable sodium metal battery based on an ionic liquid electrolyte

Sun et al.

Supplementary Figures

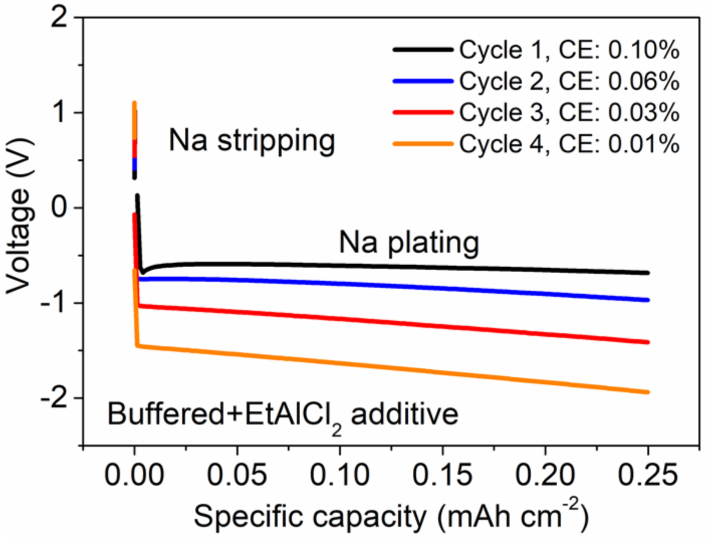

Supplementary Figure 1. Na plating/stripping profiles of a Na/Pt cell using buffered Na-Cl-IL electrolyte without [EMIm]FSI additive at a current density of 0.5 mA cm^{-2}.

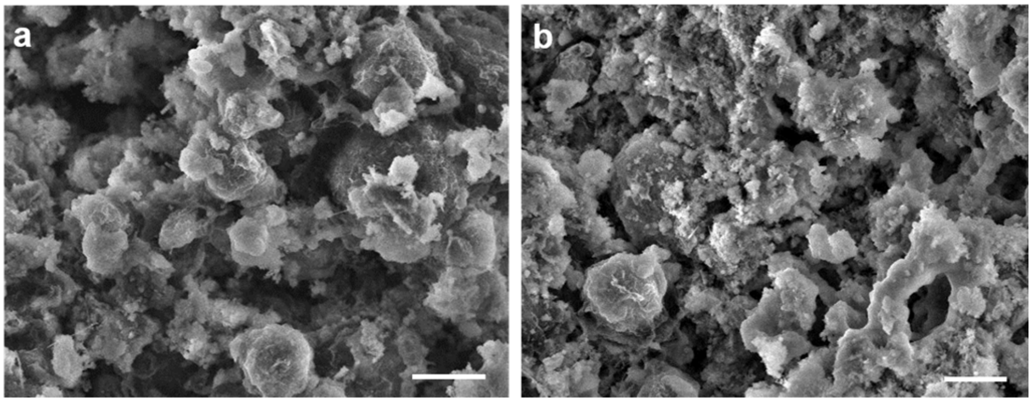

Supplementary Figure 2. Morphology of Na plating at different current densities. a, b, SEM images of Na-plated Cu foils in Na/Cu cells at a current density and of 0.5 and 1.5 mA cm^{-2}, respectively. Specific capacity, 0.5 mAh cm^{-2}. The cells were cycled for 5 cycles and stopped at discharge state (Na plating on Cu) prior to characterization. Scale bars in **a** and **b**, 10 μm.

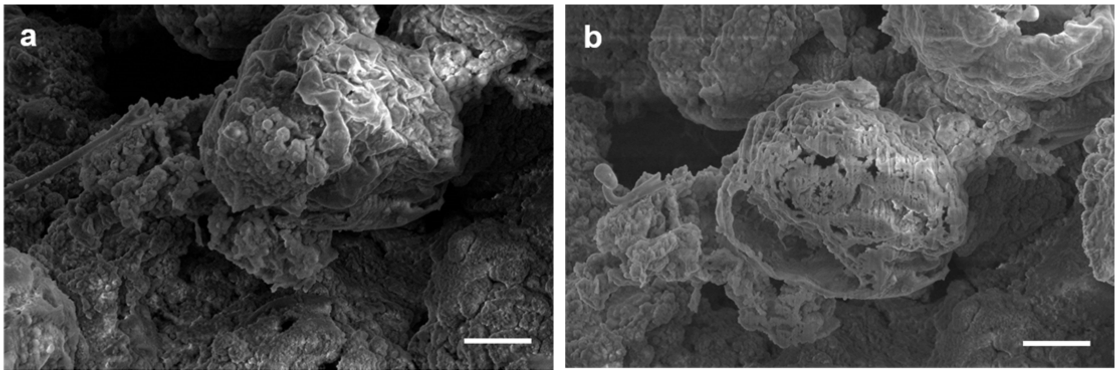

Supplementary Figure 3. Cross-section morphology of Na plating. a, b, SEM images of a Na particle before (**a**) and after (**b**) cutting via focused ion beam. Scale bars in **a** and **b**, 5 μm.

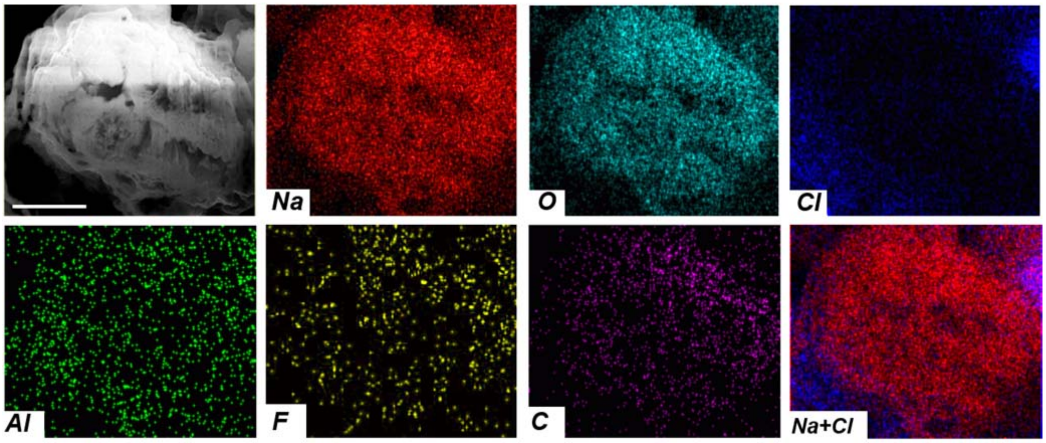

Supplementary Figure 4. SEM and the corresponded element mapping images of the cross section of a Na particle via FIB cutting. The Na particle was plated on a Cu foil at a current density of 0.5 mA cm^{-2} in a Na/Cu cell. The cell was first cycled for 10 cycles and stopped at discharge state (Na plating on Cu) prior to characterization. Scale bar, 5 μm.

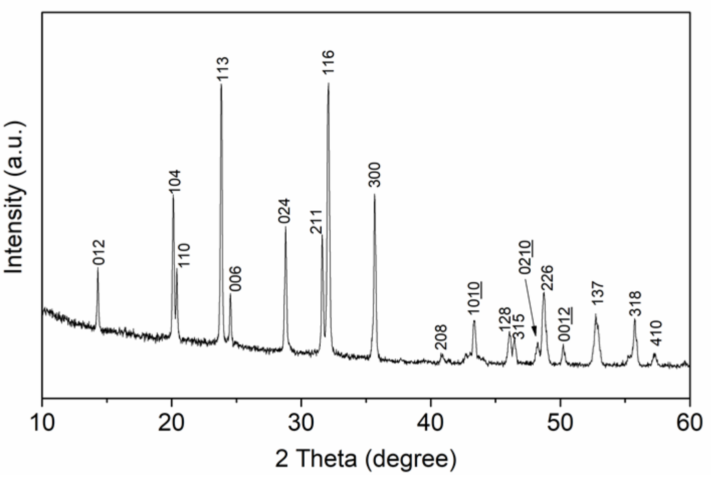

Supplementary Figure 5. XRD patterns of NVP@rGO.

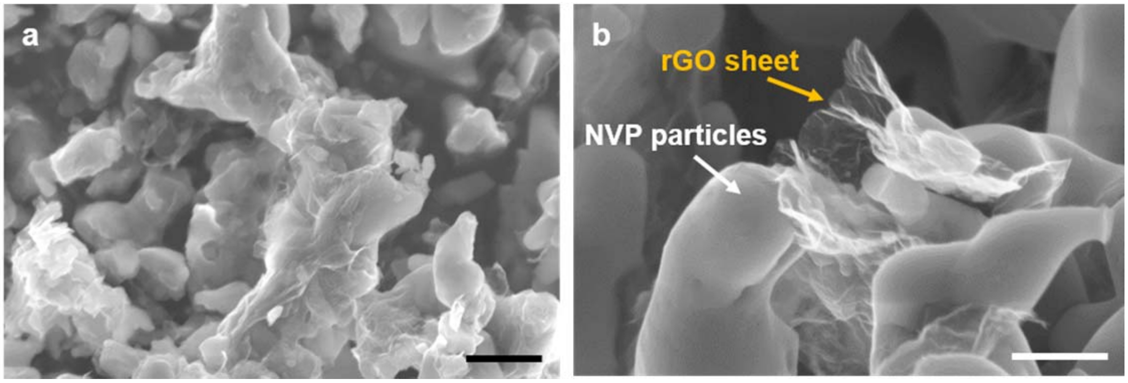

Supplementary Figure 6. Morphology of NVP@rGO. a, b, SEM images of NVP@rGO at low and high magnifications, respectively. Scale bars in **a** and **b** are 500 nm and 200 nm, respectively.

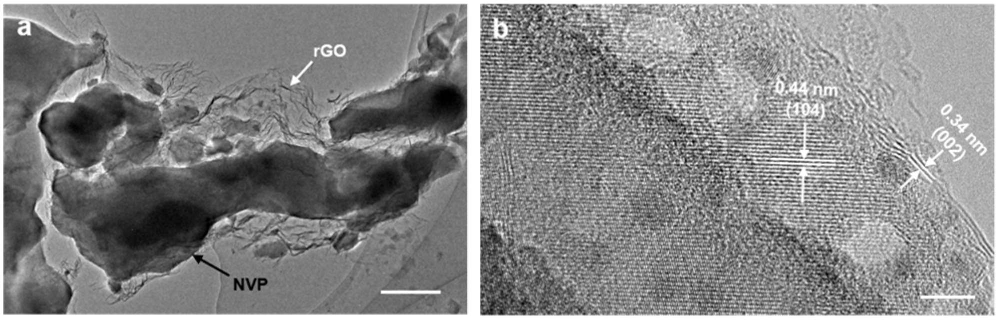

Supplementary Figure 7. TEM images of NVP@rGO. a, b, TEM and High-resolution TEM images of NVP@rGO, respectively. Scale bars in **a** and **b** are 200 nm and 5 nm, respectively.

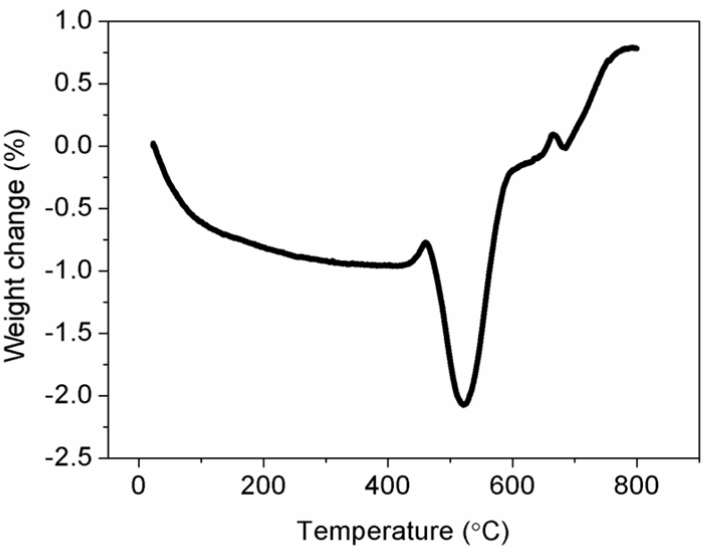

Supplementary Figure 8. TGA of NVP@rGO within a temperature range of 25-800 ^{o}C with a heating rate of 5 ^{o}C min^{-1} in air.

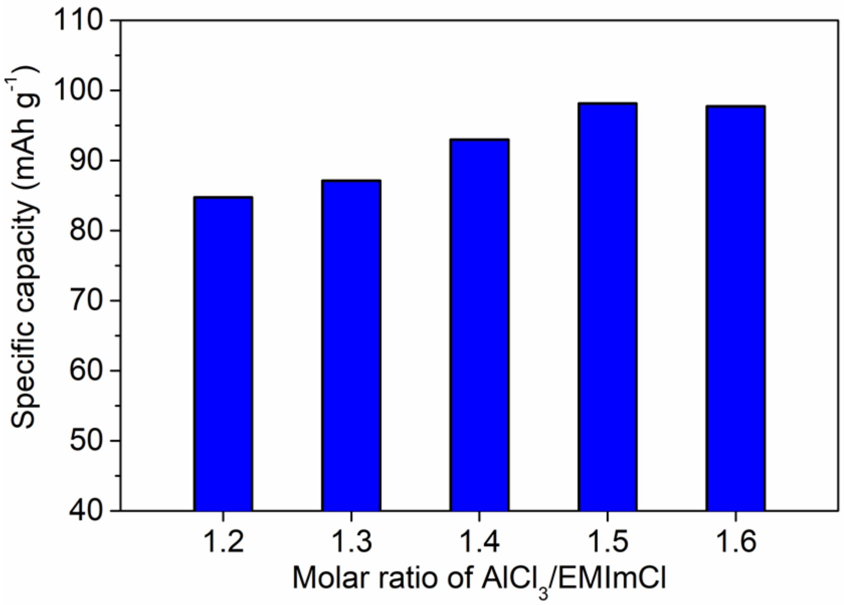

Supplementary Figure 9. Variation of specific discharge capacity of NVP@rGO on IL electrolytes with different molar ratios of $AlCl_3$ and EMIC. Current density, 25 mA g^{-1}. The specific capacity of Na-NVP@rGO battery showed a dependence with the molar ratio of $AlCl_3$/[EMIm]Cl. Increasing the molar ratio from 1.2 to 1.5 enhanced the specific capacity due to the increased Na ion concentration. However, when the molar ratio further reached 1.6, the specific capacity decreased slightly likely due to increased viscosity.

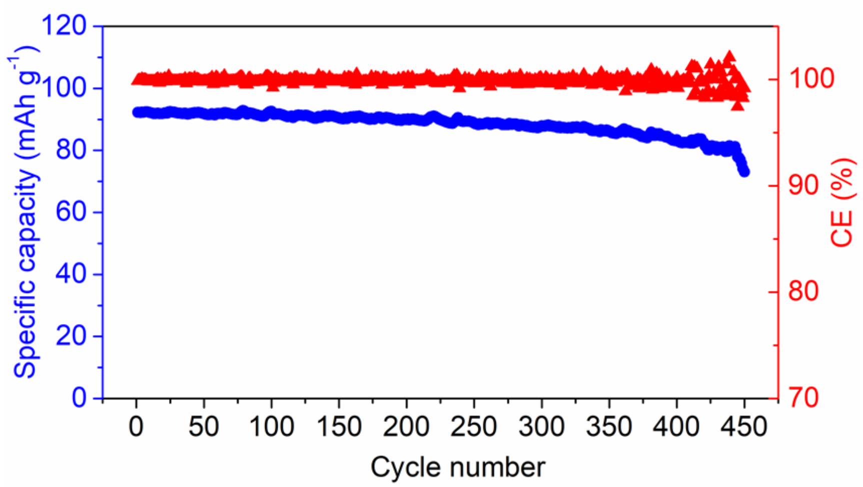

Supplementary Figure 10. Cyclic stability of a Na/NVP@rGO cell using conventional organic electrolyte consisting of 1 M $NaClO_4$ in ethylene carbonate/diethyl carbonate (EC/DEC, 1:1 by vol) with 5% FEC at a current density of 150 mA g^{-1}.

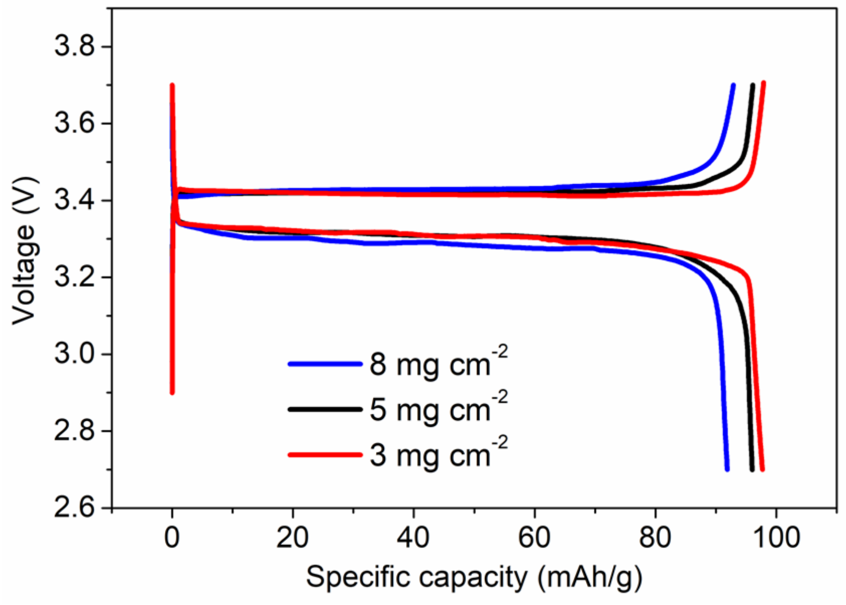

Supplementary Figure 11. Galvanostatic charge-discharge curves of Na/NVP@rGO cells with different NVP@rGO loadings of 3.0, 5.0 and 8.0 mg cm^{-2} at a current density of 25 mA g^{-1}.

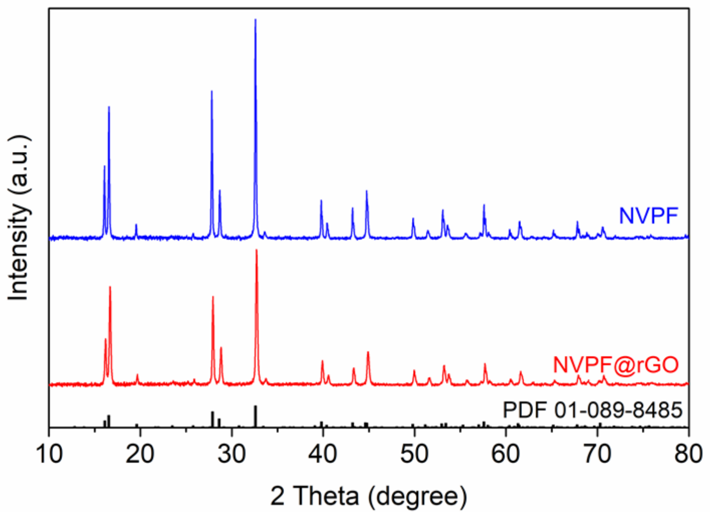

Supplementary Figure 12. XRD patterns of NVPF and NVPF@rGO.

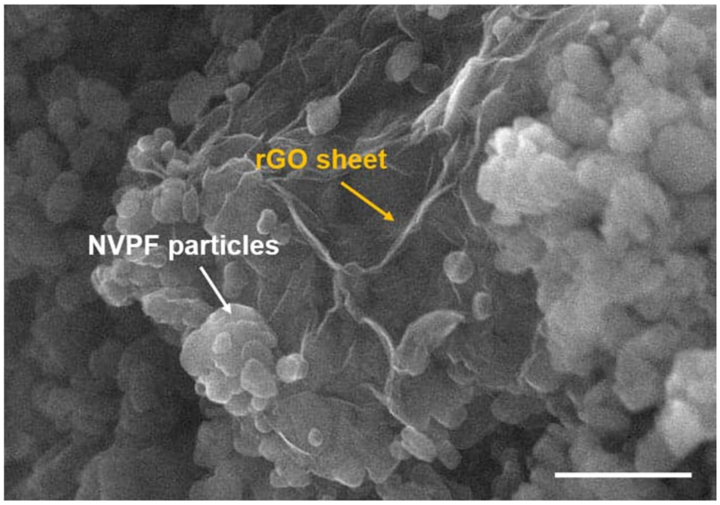

Supplementary Figure 13. SEM image of NVPF@rGO. Scale bar, 500 nm.

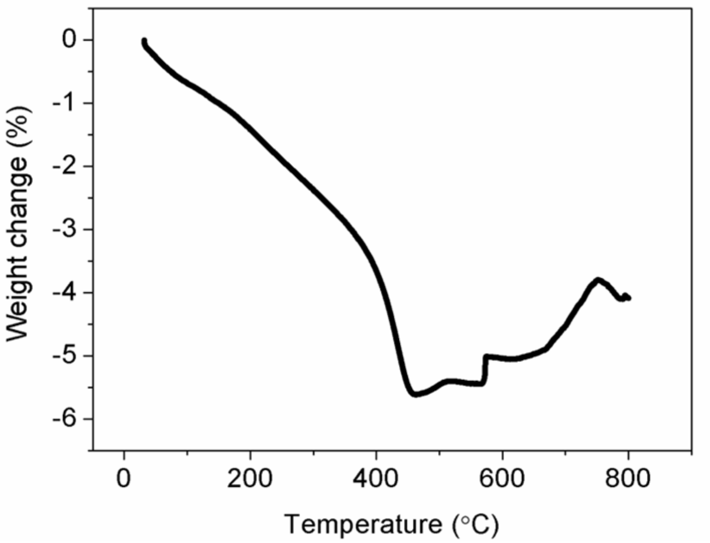

Supplementary Figure 14. TGA of NVPF@rGO within a temperature range of 25-800 ^{o}C with a heating rate of 5 ^{o}C min^{-1} in air. The temperature range used for determining rGO percentage is 180-460 ^{o}C.

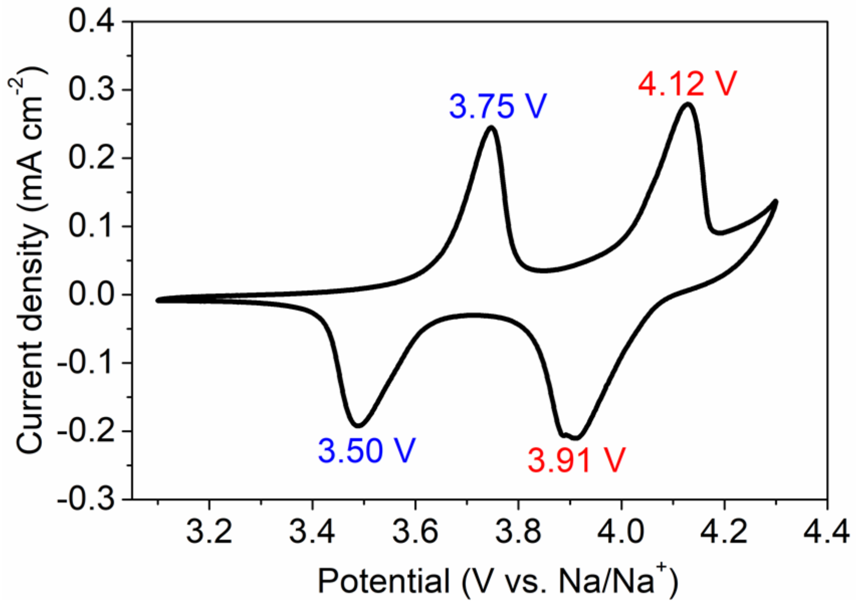

Supplementary Figure 15. CV curve of a Na/NVPF@rGO cell using Na^{+}-C-IL electrolyte at a scan rate of 0.1 mV s^{-1}.

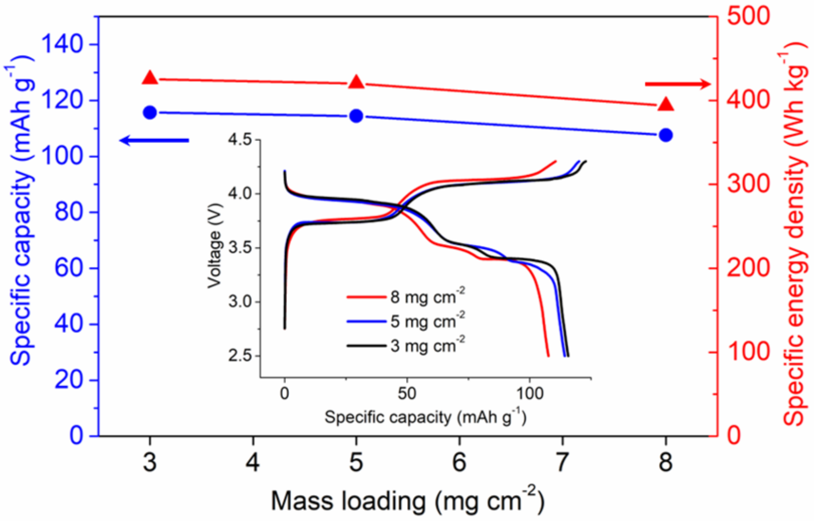

Supplementary Figure 16. Variety of specific capacity and energy density on different mass loadings from 3 to 8 mg cm^{-2}. The inset showed corresponding galvanostatic charge-discharge curves with different loadings 50 mA g^{-1}.

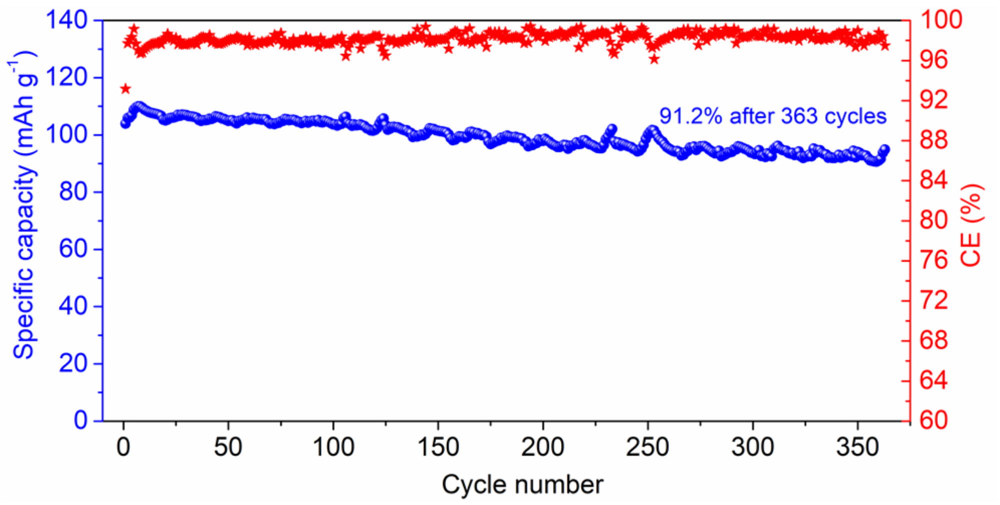

Supplementary Figure 17. Cyclic stability of a Na/NVPF@rGO cell with a NVPF@rGO mass loading of 5.3 mg cm^{-2} using buffered+$EtAlCl_2$/[EMIm]FSI additive IL electrolyte. Current density, 150 mA g^{-1}.

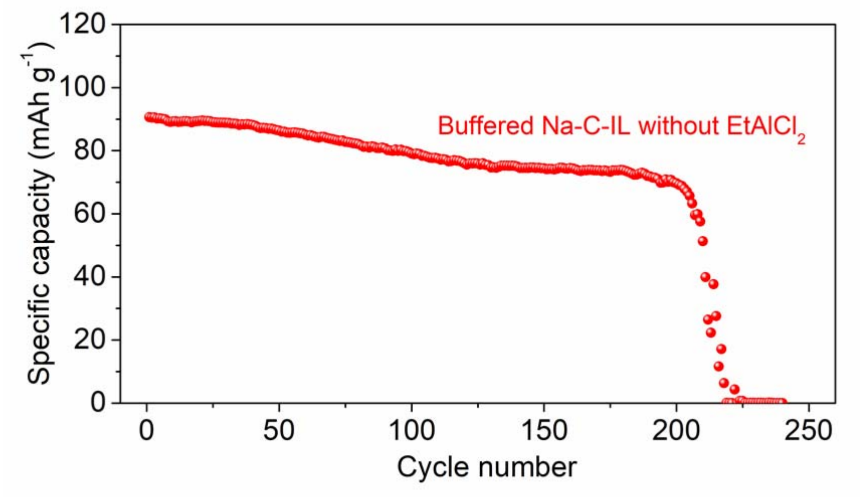

Supplementary Figure 18. Cyclic stability of a Na/NVPF@rGO cell using buffered Na^{+}-C-IL electrolyte without $EtAlCl_2$ additive at 150 mA g^{-1} for 300 cycles. The mass loading of NVPF@rGO was 3.0 mg cm^{-2}.

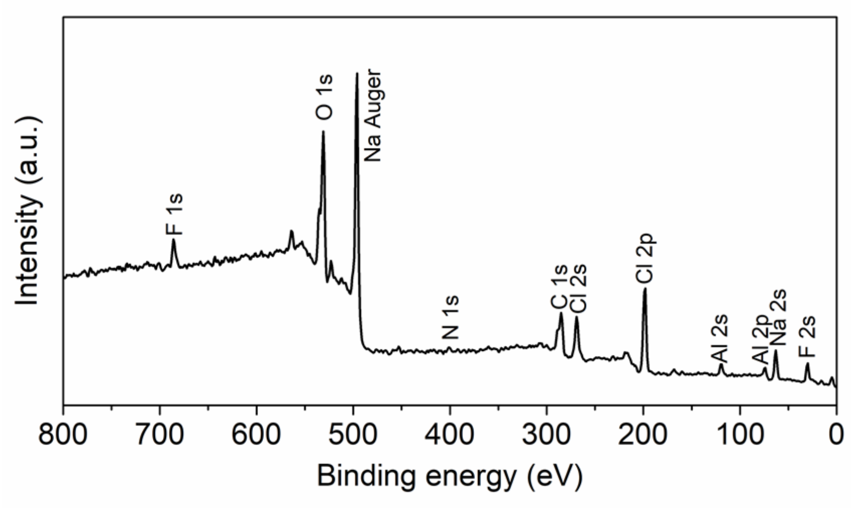

Supplementary Figure 19. Surface XPS spectrum of a Na anode from a Na/NVP@rGO cell with the NVP@rGO mass loading of 5.0 mg cm^{-2} at fully charged state. Prior to XPS measurement, the cell was cycled for 20 cycles at 100 mA/g for sufficient formation of SEI.

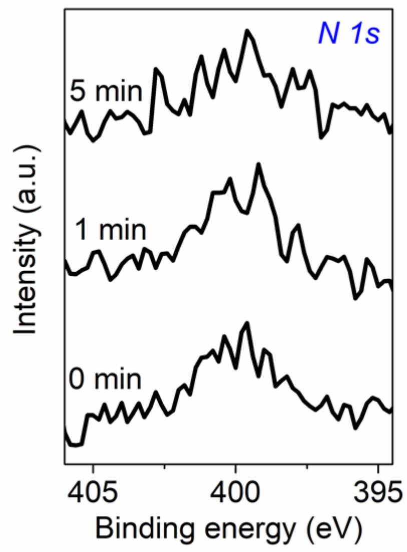

Supplementary Figure 20. High-resolution XPS spectra for N 1s of the Na anode from a Na/NVP@rGO cell with the NVP@rGO mass loading of 5.0 mg cm^{-2} at different depths. Prior to XPS measurement, the cell was cycled for 20 cycles at 1 C for sufficient formation of SEI.

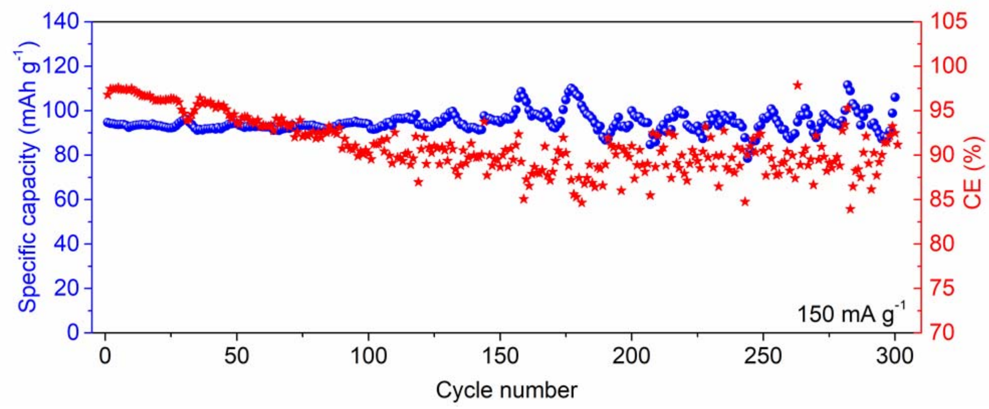

Supplementary Figure 21. Capacity and Colombic efficiency retention of a Na/NVP@rGO cell using 1 M NaFSI in [EMIm]FSI IL electrolyte at 150 mA g^{-1}.

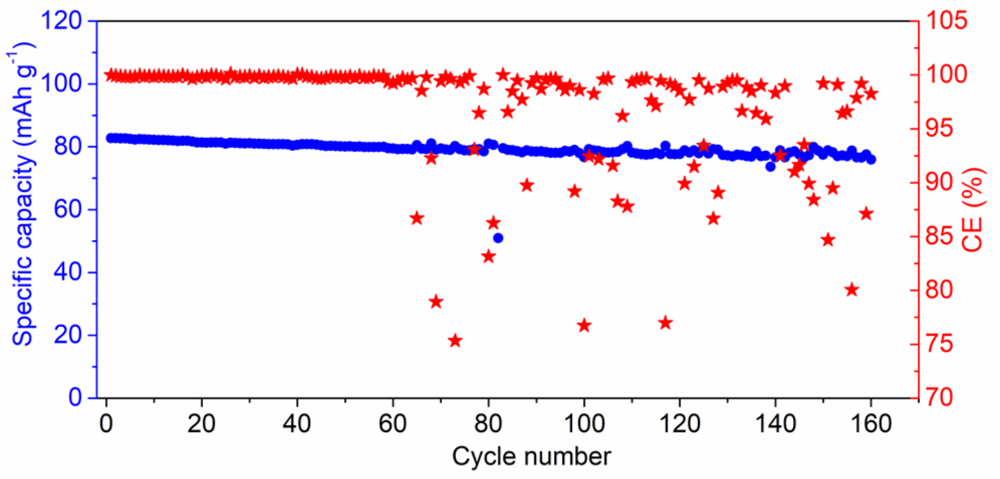

Supplementary Figure 22. Capacity and Colombic efficiency retention of a Na/NVP@rGO cell using NaFSI/N-propyl-N-methylpyrrolidinium bis(fluorosufonyl)imide (molar ratio of 2:8) IL electrolyte. Current density, 150 mA g^{-1}.

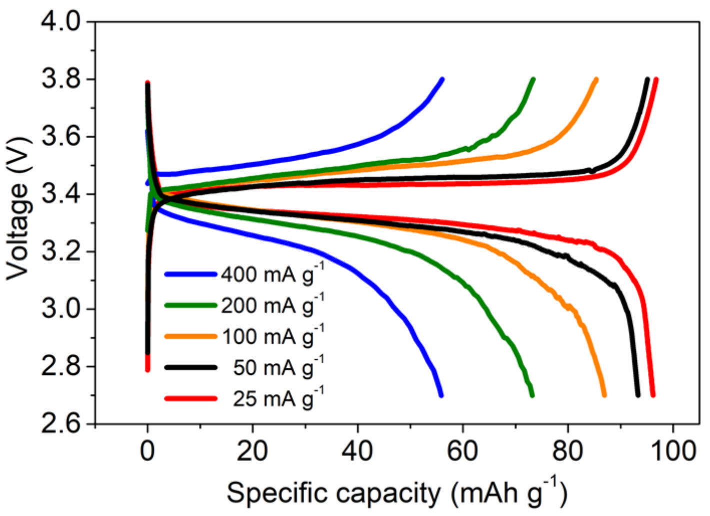

Supplementary Figure 23. Galvanostatic charge-discharge curves of a Na/NVP@rGO cell using NaFSI/N-propyl-N-methylpyrrolidinium bis(fluorosufonyl)imide (molar ratio of 2:8) IL electrolyte at varied current densities from 25 to 400 mA g^{-1}.

Supplementary Tables

Supplementary Table 1. Comparison of representative Na metal battery performances based on IL electrolytes.

Cathode	Electrolyte	C_{sp} (mAh g^{-1})	CE	Cyclic stability	Loading (mg cm^{-2})	Discharge voltage (V)	E (Wh kg^{-1})	P (W kg^{-1})	Ref.
$NaFePO_4$	1 M $NaBF_4$ in BMP-TFSI	92	-	67%@100 cycles	~ 1.9	~ 2.8	~ 253	~ 66	1
$NaFePO_4$	1 M $NaClO_4$ in BMP-TFSI	79	-	62%@100 cycles	~ 1.9	~ 2.8	~ 217	~ 34	1
$NaFePO_4$	1 M $NaPF_6$ in BMP-TFSI	44	-	57%@100 cycles	~ 1.9	~ 2.8	~ 121	~ 12	1
$Na_{0.44}MnO_2$	1 M $NaBF_4$ in BMP-TFSI	84	-	40%@100 cycles	-	~ 2.7	~ 227	~ 28	1
$Na_{0.44}MnO_2$	1 M $NaClO_4$ in BMP-TFSI	97	-	65%@100 cycles	-	~ 2.7	~ 262	~ 52	2
$Na_{0.44}MnO_2$	1 M NaTFSI in BMP-TFSI	92	-	50%@100 cycles	-	~ 2.7	~ 248	~ 37	2
$Na_{0.44}MnO_2$	1 M $NaPF_6$ in BMP-TFSI	38	-	33%@100 cycles	-	~ 2.7	~ 103	~ 9	2
NVP@C	1 M NaFSI in $Py_{13}FSI$	89	99%	~99%@50cycles	-	~ 3.3	~ 294	~ 1620	3
$Na_{0.6}Ni_{0.22}Fe_{0.11}Mn_{0.66}O_2$	0.2 M NaTFSI in $Py_{14}TFSI$	~ 150	98%	~ 93%@5 cycles	1.0	~ 3	~ 450	~ 30	4
$Na_{0.45}Ni_{0.22}Co_{0.11}Mn_{0.66}O_2$	0.45 M NaTFSI in $Py_{14}TFSI$	~ 220	-	~80%@100 cycles	~ 2.5	2.7	550	~ 30	5
$NaCrO_2$	NaFSI in $Py_{13}FSI$ (2: 8 in molar)	92	99.7%	-	-	~ 2.9	~ 267	~ 64	6
NVP@rGO	Buffered Na-Cl-IL	98	99.9%	~ 96%@460 cycles	3.0	~ 3.3	323	~ 1650	This work
NVPF@rGO	Buffered Na-Cl-IL	114	99.0%	~ 90.6%@710 cycles	3.0	~3.75	~ 420	~ 1766	This work

C_{sp}, CE, E and P represent specific discharge capacity, Coulombic efficiency, energy density and power density, respectively. BMP, *N*-butyl-*N*-methylpyrrolidinium. Py_{13},

N-methyl-*N*-propylpyrrolidinium. Py_{14}, N-butyl-N-methylpyrrolidinium. FSI, bis(fluorosulfonyl)imide. TFSI, bis(trifluoromethanesulfonyl)imide.

Supplementary Methods

Preparation of graphene oxide. 1 g flake graphite powder was pre-oxidized in the mixture of 30 mL sulfuric acid and 10 mL nitric acid under stirring for 24 h. After washing with deionized water and drying, the obtained powder was exfoliated in a tube furnace at 1000 °C for 10 s, followed by reacting with 60 mL oleum, 0.84 g $K_2S_2O_8$ and 1.3 g P_2O_5 at 80 °C for 5 h under stirring. After cooling down to room temperature, 500 mL deionized water was slowly added to the suspension, and the dried products were obtained by vacuum filtrating and washing for 3 times, and dried in a vacuum oven. The resulted powder was added to 50 mL oleum in ice bath, followed by adding 3 g $KMnO_4$ slowly under vigorous stirring, during which the temperature was kept below 20 °C. The mixture was then heated to 35 °C and stirred for another 2 h, and diluted with 500 mL deionized water and added with 2 mL 30 wt% H_2O_2. The dispersion was left overnight, and the brown gel at bottom was washed with deionized water, followed by centrifuging with 1 M HCl solution for 5 times, and then washing with deionized water until the decantate turned nearly neutral.

Details of battery assembly and testing. We found that the powders of NVP@rGO and NVPF@rGO are best to store in an Ar-filled glove box to avoid possible contaminations and absorption of moisture in air. Freshly prepared NVP@rGO and NVPF@rGO electrodes are preferable for good battery performances. Sufficient contact between electrode and separator is important for good rate and cycling performances. The pouch cell was placed under vacuum for 15 min after injecting the electrolyte to enhance the electrolyte permeation into separator and electrodes. The edges of the pouch cells were flattened, and the pouch was further clamped using two clips (0.75 inch, Clipco) between two hardboards for 30 min, realizing a good contact between the electrode and separator. The clips were then removed and no extra pressure was applied on the battery during testing.

Supplementary References

1. Wongittharom, N., Wang, C.-H., Wang, Y.-C., Yang, C.-H. & Chang, J.-K. Ionic Liquid Electrolytes with Various Sodium Solutes for Rechargeable Na/NaFePO4 Batteries Operated at Elevated Temperatures. *ACS Appl. Mater. Interfaces* **6**, 17564–17570 (2014).
2. Wang, C.-H. *et al.* Rechargeable Na/$Na_{0.44}MnO_2$ cells with ionic liquid electrolytes containing various sodium solutes. *J. Power Sources* **274**, 1016–1023 (2015)
3. Manohar, C. V. *et al.* Ionic liquid electrolytes supporting high energy density in sodium-ion batteries based on sodium vanadium phosphate composites. *Chem. Commun.* **54**, 3500–3503 (2018).
4. Hasa, I., Passerini, S. & Hassoun, J. Characteristics of an ionic liquid electrolyte for sodium-ion batteries. *J. Power Sources* **303**, 203–207 (2016).
5. Chagas, L. G., Buchholz, D., Wu, L., Vortmann, B. & Passerini, S. Unexpected performance of layered sodium-ion cathode material in ionic liquid-based electrolyte. *J. Power Sources* **247**, 377–383 (2014).
6. Ding, C. *et al.* NaFSA-C1C3pyrFSA ionic liquids for sodium secondary battery operating over a wide temperature range. *J. Power Sources* **238**, 296–300 (2013).
