# Supplementary material for: A safe and non-flammable sodium metal battery based on an ionic liquid electrolyte
Source: Nat Commun. 2019 Jul 24;10:3302. doi: 10.1038/s41467-019-11102-2 (PMC6656735; doi:10.1038/s41467-019-11102-2)
Supplement: Supplementary file 3 — Description of Additional Supplementary Files [file 41467_2019_11102_MOESM3_ESM.pdf]

Description of Additional Supplementary Files

File Name: Supplementary Movie 1
Description: Buffered Na-Cl-IL electrolyte was soaked into a glass fibre separator, contacted with flame and did not catch fire, which confirmed the non-flammable nature of the IL electrolyte.

File Name: Supplementary Movie 2
Description: Conventional organic carbonate electrolyte, i.e., 1.0 M $NaClO_4$ in ethylene carbonate/diethyl carbonate (1:1 by vol) with 5 wt% FEC was soaked into a glass fibre separator and contacted with flame. The flammable electrolyte easily caught fire and burned immediately.
